# Triple Diuretics and Aquaretic Strategy for Acute Decompensated Heart Failure due to Volume Overload

**DOI:** 10.1155/2013/750794

**Published:** 2013-12-30

**Authors:** Rita Jermyn, Naveed Rajper, Chelsea Estrada, Sagar Patel, Michelle Weisfelner Bloom, Nand K. Wadhwa

**Affiliations:** ^1^Division of Cardiology, Department of Medicine, Stony Brook Medicine, Stony Brook, NY 11794, USA; ^2^Department of Medicine, Stony Brook Medicine, Stony Brook, NY 11794, USA; ^3^Division of Nephrology, Department of Medicine, Stony Brook Medicine, Stony Brook, NY 11794, USA

## Abstract

Diuretics, including furosemide, metolazone, and spironolactone, have historically been the mainstay of therapy for acute decompensated heart failure patients. The addition of an aquaretic-like vasopressin antagonist may enhance diuresis further. However, clinical experience with this quadruple combination is lacking in the acute setting. We present two hospitalized patients with acute decompensated heart failure due to massive fluid overload treated with a combination strategy of triple diuretics in conjunction with the aquaretic tolvaptan. The first patient lost 72.1 lbs. (32.7 kg) with an average urine output of 3.5 to 7.5 L/day over eight days on combined therapy with furosemide, metolazone, spironolactone, and tolvaptan. The second patient similarly achieved a weight loss of 28.2 lbs. (12.8 kg) over 4 days on the same treatment. Both patients maintained stable serum sodium, potassium, and creatinine over this period and remained out of the hospital for more than 30 days. Thus, patients hospitalized with acute decompensated heart failure due to volume overload can achieve euvolemia rapidly and without electrolytes disturbances using this regimen, while being under the close supervision of a team of cardiologists and nephrologists. Additionally, this therapy can potentially decrease the need for ultrafiltration and the length of hospital stay.

## 1. Introduction

Patients with congestive heart failure decompensate mainly because of enhanced sympathetic, arginine vasopressin, and renin-angiotensin-aldosterone activation [1]. This neurohumoral activation leads to upregulation of the renin-angiotensin system and intrarenal vasoconstriction which accelerates renal sodium and water retention leading to volume overload [2]. Diuretics have historically been the mainstay of therapy during heart failure admissions [3, 4]. Tolvaptan has been utilized to improve fluid and osmotic balance by inhibiting water retention without altering electrolytes in acute decompensated heart failure (ADHF) [5–7]. However, if the patient concurrently has a marked reduction in their glomerular filtration rate, diuretic resistance becomes more probable. In oliguric and anuric ADHF patients with severe volume overload and need for aggressive diuresis, ultrafiltration is a successful treatment strategy for achieving optimal volume control [8]. The safe use of tolvaptan in combination with diuretics has been reported in patients with decompensated heart failure due to volume overload [9–11]. We developed a combination protocol of furosemide, metolazone, spironolactone, and tolvaptan to augment volume loss by increasing urine output while maintaining electrolyte stability in the intravascular space before considering ultrafiltration. Here, we report our experience in two hospitalized cardiorenal patients with ADHF due to massive volume overload treated with a triple diuretics and aquaretic therapy consisting of furosemide, metolazone, spironolactone, and tolvaptan under close monitoring of serum electrolytes.

## 2. Case 1

**Figure 1:**
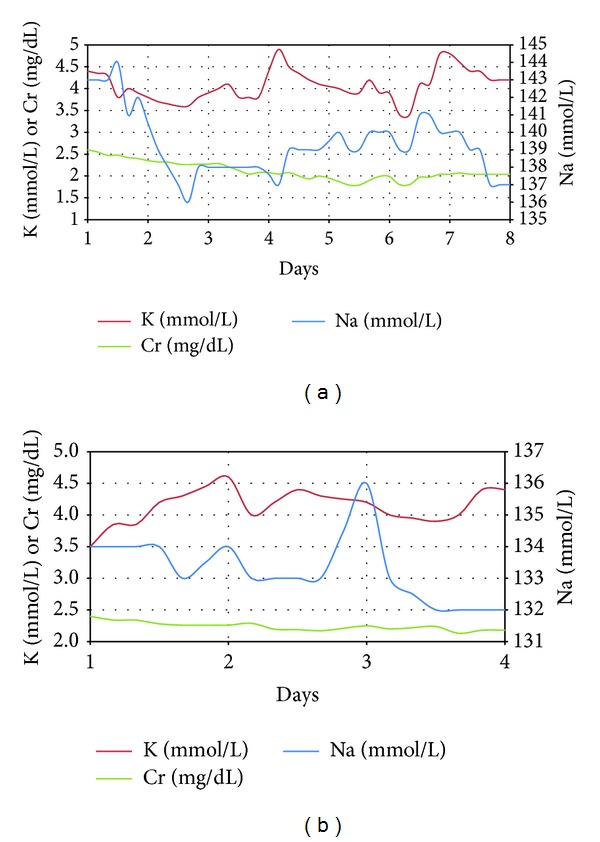
Change in serum potassium, sodium, and creatinine during treatment with quadruple therapy in Case 1 (a) and Case 2 (b).

A 67-year-old white man presented to the emergency room with progressive dyspnea and along with a weight gain of 20 lbs. (9.1 kg) within three weeks. Cardiac history was significant for a dilated ischemic cardiomyopathy (New York Heart Association class III), status after revascularization with coronary artery bypass grafting (CABG) and stents, left ventricular ejection fraction (LVEF) of 35%, severe diastolic dysfunction, and chronic kidney disease (CKD) stage 4 due to cardiorenal syndrome. On physical examination, the patient was afebrile and in mild respiratory distress, with difficulty speaking full sentences. His blood pressure was 104/71 mmHg, heart rate was 55/min, respiratory rate was 18/min, and oxygen saturation was 92% on 4 L nasal cannula. His weight was 159.2 kilograms. He had jugular venous distension to the angle of the mandible. Cardiac exam revealed regular rhythm with an S3 gallop. Lung exam showed bilateral rales. Abdomen was protuberant with ascites. Laboratory data revealed serum sodium of 137 mmol/L (mEq/L), potassium of 5.2 mmol/L (mEq/L), creatinine of 2.5 mg/dL (221 *μ*mol/L), chloride of 101 mmol/L (mEq/L), and bicarbonate of 30 mmol/L (mEq/L). His B-type natriuretic peptide was 456 pg/mL (ng/L). Chest roentgenogram showed large right and moderate left pleural effusions. He was treated with a triple diuretics and aquaretic regimen (Table 1) to optimize his severe volume overload. Over the course of eight days with this combination therapy, the patient achieved a 72.1 lbs. (32.7 kg) weight loss. His serum creatinine improved to 2.12 mg/dL (187.4 *μ*mol/L), while his serum electrolytes remained stable as shown in Figure 1(a). This regimen expedited weight loss with resolution of dyspnea. The patient successfully remained out of the hospital for over thirty days.

## 3. Case 2

A 79-year-old white man presented to the emergency room with a weight gain of 15 lbs. (6.8 kg) over two weeks associated with progressive bilateral pitting edema and paroxysmal nocturnal dyspnea. His medical history included a non-dilated ischemic cardiomyopathy with LVEF of 30% (New York Heart Association class III), status-post CABG, status-post biventricular automatic implantable cardioverter defibrillator (AICD), and CKD stage 4 due to cardiorenal syndrome. Physical examination revealed temperature of 37.1 C, blood pressure of 103/55 mmHg, heart rate of 68/min, respiratory rate of 17/min, and oxygen saturation of 96% in room air. His weight was 88.8 kilograms. He had jugular venous distention. Cardiac exam revealed regular S1/S2 with a II/VI SEM radiating to left lateral axilla. His lung exam revealed bibasilar rales. His abdomen was markedly distended with ascites. The lower extremities had gross pitting edema up to the waist. His serum sodium was 130 mmol/L (mEq/L), potassium was 5.1 mmol/L (mEq/L), bicarbonate was 29 mmol/L (mEq/L), and creatinine was 2.5 mg/dL (221 *μ*mol/L), with chloride of 100 mmol/L (mEq/L) and B-type natriuretic peptide of 442 pg/mL (ng/L). Electrocardiogram showed no signs of acute ischemia. Chest roentgenogram showed pulmonary vascular congestion. He was managed with the triple diuretics and aquaretic regimen (Table 1) for ADHF. Over four days, the patient achieved a 28.2 lb. (12.8 kg) weight loss. His serum creatinine improved to 2.18 mg/dL (192.7 *μ*mol/L) with stable serum electrolytes as shown in Figure 1(b). He remained out of the hospital for over thirty days.

## 4. Discussion

We report two patients with ADHF due to severe volume overload and cardiorenal syndrome, who achieved clinical euvolemic status with the diuretics and aquaretic regimen including furosemide, metolazone, spironolactone, and tolvaptan within one week's time. Their renal function and serum electrolytes remained stable, and both patients stayed out of the hospital for more than thirty days. With frequent readmissions and lengthy hospitalizations for acute decompensated heart failure, this strategy might expedite restoration to a clinically euvolemic state and decrease thirty-day hospital readmission rates.

Ross et al. evaluated trends in readmission after ADHF hospitalization and found that 30-day readmission rates from 2004 to 2006 ranged from 23.7% to 23.9% [12]. This suggests that newer approaches to treatment are much needed. Diuretics remain the mainstay of therapy for patients with ADHF. In the Diuretic Strategies in Patients with Acute Decompensated Heart Failure (DOSE) trial, Felker et al. noted no significant difference in patients' assessment of symptoms when the diuretic furosemide was administered by either bolus or continuous infusion [4]. A combination pharmacologic therapy using spironolactone, furosemide, and metolazone has been well described to improve diuresis [13]. However, in patients unable to achieve effective diuresis, ultrafiltration is viewed as an alternative regimen [8]. In the ultrafiltration versus intravenous diuretics for patients hospitalized for acute decompensated heart failure (UNLOAD) trial, ultrafiltration produced a greater weight loss at 48 hours (5.0 kg) compared to usual care (3.3 kg) (*P* = 0.001) [14]. However, a more recent trial suggested that in hospitalized patients with decompensated heart failure, the use of pharmacotherapy was superior to ultrafiltration for preserving renal function and achieving weight loss with fewer adverse events [15]. A stepped-up pharmacologic care utilized loop and thiazide diuretics as well as inotropes and intravenous vasodilators [15]. Given the limitations of current available therapies, it seemed reasonable to look for additional strategies using medical therapy. Efficacy and safety of tolvaptan with the concurrent use of diuretics have been reported in patients with decompensated heart failure due to volume overload [7, 9–11]. Tolvaptan as an additional therapy in ADHF patients also prevents hyponatremia which is seen with loop diuretics [6]. Therefore, we constructed a protocol with a combination of diuretics and aquaretic therapy to achieve rapid diuresis safely in hospitalized patients with ADHF and cardiorenal syndrome. Given the mechanisms of action of these drugs, we utilized their unique pharmacokinetic and pharmacodynamics properties to achieve rapid and effective diuresis while closely monitoring serum electrolytes and renal function. In the literature, it is well described that the therapeutic effect of diuretics is similar in nonischemic and ischemic cardiomyopathy, but response to some other drugs such as antiarrhythmics is better in nonischemic heart failure [16].

In conclusion, we describe a successful strategy in treating grossly volume overloaded acute decompensated heart failure patients, achieving high urine output while maintaining stable serum electrolytes and creatinine, and reducing 30-day hospital readmission rates. We recommend that this protocol should only be used in the cardiac intensive care unit setting with close monitoring of serum electrolytes under the supervision of an experienced team of cardiologists and nephrologists.

## Figures and Tables

**Table 1:** Diuretics' protocol: doses of furosemide and metolazone were titrated based on urine output; spironolactone dose was titrated to maintain a serum potassium of 4–4.5 mmol/L (mEq/L); tolvaptan dose was titrated to maintain a serum sodium of 131–139 mmol/L (mEq/L) (based on the initial value, maximum correction was 6–8 mmol/L (mEq/L) and not to exceed 139 mmol/L (mEq/L) in 24 hours).

Diuretics and suggested doses	Laboratory monitoring	Serum electrolyte goals	Target fluid loss
Furosemide: infusion 10–40 mg/hr	Serum chemistry every 6 hours	Sodium: 131–139 mmol/L (mEq/L)	4-5 liters/day
Spironolactone: 25 mg daily to 50 mg three times daily	Urine electrolytes every 6 hours	Potassium: 4–4.5 mmol/L (mEq/L)	
Metolazone: 5 mg twice daily to 10 mg twice daily			
Tolvaptan: 15 mg daily to 30 mg daily			
